# Comparative efficacy and safety of oral Chinese patent medicines combined with conventional therapy for coronary heart disease complicated by diabetes: a systematic review and network meta-analysis

**DOI:** 10.3389/fcvm.2026.1785694

**Published:** 2026-04-30

**Authors:** Dan Han, Bo-yi Wang, Xin-tian Yu, Chong-chai Li, Bo Liu, Zhan-ze Ma

**Affiliations:** 1 Department of Integrated Traditional Chinese and Western Medicine, Liaoning University of Traditional Chinese Medicine Xinglin College, Shenyang, China; 2 Organization Department, Liaoning University of Traditional Chinese Medicine, Shenyang, China; 3 Department of Integrated Traditional Chinese and Western Medicine, Liaoning Cancer Hospital, Shenyang, China; 4 Admissions and Employment Office, Liaoning University of Traditional Chinese Medicine, Shenyang, China

**Keywords:** comorbidity, coronary heart disease, diabetes mellitus, network meta-analysis, oral Chinese patent medicine

## Abstract

**Background:**

Coronary heart disease (CHD) frequently coexists with diabetes mellitus (DM), resulting in a complex metabolic–vascular disorder with poor cardiovascular prognosis. Oral Chinese patent medicines (OCPMs) are widely used as adjunctive therapies in China. However, their comparative efficacy and safety remain unclear.

**Methods:**

We systematically searched Chinese and international databases for randomized controlled trials (RCTs) evaluating OCPMs combined with conventional Western therapy in patients with CHD complicated by DM. We conducted a network meta-analysis using R and Stata software. The evaluated outcomes included glycemic control, lipid profiles, cardiac function, angina-related measures, inflammatory markers, endothelial function, hemorheological parameters, major adverse cardiovascular events (MACEs), and adverse reactions. We assessed treatment rankings using the surface under the cumulative ranking curve.

**Results:**

A total of 71 RCT articles were included, with a total sample size of 7,988 patients, involving 14 types of OCPMs. The NMA revealed the following results: Shexiang Baoxin Pills combined with conventional Western medicine exhibited optimal efficacy in reducing triglycerides (TG), improving high-density lipoprotein cholesterol (HDL-C) and left ventricular end-systolic diameter (LVESD), decreasing the weekly frequency and duration of angina pectoris episodes, and lowering levels of interleukin-6 (IL-6), tumor necrosis factor-α (TNF-α), high-sensitivity C-reactive protein (hs-CRP), endothelin-1 (ET-1), and fibrinogen (FIB). Tongxinluo Capsules combined with conventional Western medicine achieved the best outcomes in clinical efficacy, reducing 2-h postprandial blood glucose (2 h PBG), improving left ventricular end-diastolic diameter (LVEDD), increasing nitric oxide (NO), and improving malondialdehyde (MDA). Yindan Xinnaotong Soft Capsules combined with conventional Western medicine showed the most prominent effects in enhancing left ventricular ejection fraction (LVEF), reducing whole blood high-shear viscosity and low-shear viscosity, and improving superoxide dismutase (SOD). Guanxinning Tablets combined with conventional Western medicine demonstrated optimal efficacy in lowering glycated hemoglobin (HbA1c). Jinlida Granules combined with conventional Western medicine yielded the best results in reducing fasting blood glucose (FBG) and low-density lipoprotein cholesterol (LDL-C). Liuwei Dihuang Pills combined with conventional Western medicine achieved optimal efficacy in reducing the incidence of MACE. Yangxinshi Tablets combined with conventional Western medicine exhibited the most significant decrease in N-terminal pro-B-type natriuretic peptide (NT-proBNP). Finally, Danzhi Jiangtang Capsules combined with conventional Western medicine achieved the best outcomes in reducing total cholesterol (TC).

**Conclusions:**

OCPMs combined with conventional therapy provide multidimensional benefits for patients with CHD complicated by diabetes. However, the findings should be interpreted with caution due to heterogeneity and variable study quality, highlighting the need for high-quality, large-scale, multicenter RCTs to confirm these results.

**Systematic Review Registration:**

PROSPERO CRD42024626573.

## 1 Introduction

Coronary heart disease (CHD) remains the leading cause of cardiovascular morbidity and mortality worldwide, primarily driven by atherosclerotic coronary artery narrowing and myocardial ischemia. With the increasing prevalence of metabolic disorders, diabetes mellitus (DM) has emerged as one of the most important comorbidities affecting patients with CHD. Epidemiological studies (1, 2) indicate that nearly one-third of patients with CHD exhibit impaired glucose metabolism, while the presence of diabetes substantially increases the risks of hospitalization, major adverse cardiovascular events (MACE), and cardiovascular mortality. Importantly, diabetes not only accelerates atherosclerosis progression but also exacerbates myocardial ischemia and heart failure, underscoring a synergistic rather than additive interaction between the two diseases (3).

**Table 1:** Basic characteristics of included studies

**Study**	**Sample size (n)**		**Age (years)**		**Disease duration (years)**		**Treatment group intervention**	**Treatment course**	**Outcome measures**
	**T**	**C**	**T**	**C**	**T**	**C**			
Li (11)	30	30	–	–	8.01±1.72	8.23±1.48	GXXKAC+CT	1 month	②
Li and Du (12)	30	30	55.17±7.41	62.8±11.7	12.5±7.5	10.5±5.5	GXXKAC+CT	4 weeks	①②③
Meng et al. (13)	60	60	61.2±8.8	61.8±9.2	5.8±2.2	5.6±2.8	LWDHP+insulin+CT	3 months	②③㉕
Yang et al. (14)	172	171	57.93±9.77	58.63±10.27	–	–	TXLC+CT	1 year	⑳㉑㉕
Zhang et al. (15)	40	40	60.9 ± 7.6	61.2 ± 7.4	–	–	TXLC+CT	12 weeks	⑤⑥⑦⑧⑨
Li (16)	50	50	53–57	51–75	2–6	2–5	YXST+CT	6 months	①②③④⑫⑰㉖
Meng et al. (17)	55	54	55.4±6.8	54.3±7.9	–	–	FFDSDP+insulin+CT	8 weeks	②③⑮⑯⑰
Wu (18)	37	37	65.23±8.46	64.56±8.77	–	–	FFDSDP+CT	1 year	②③④㉕
Sun et al. (19)	40	40	72. 6± 2. 6	71. 9±2. 5	5. 7±0. 5	5. 9±0. 4	LWDHP+CT	3 months	②㉕
Qin et al. (20)	45	45	64.2 ± 8.5	65.3 ±7.9	8.2 ±3.5	8.1 ± 4.1	QSYQDP+CT	8 weeks	①②⑤⑥⑦⑧
Zhang (21)	60	60	62.8±5.1	62.5±4.8	8.3±7.5	8.1±7.8	SXBXP+trimetazidine+CT	8 weeks	①⑤⑥⑦⑧㉔
Yu et al. (22)	24	24	70.12±8.13	70.28±8.31	4.01±1.15	4.12±1.36	SXBXP+CT	3 weeks	②③⑬⑭
Zheng (23)	37	37	70.24±2.85	71.47±3.04	5.92±2.21	5.58±2.63	SXBXP+CT	1 month	①③
Wang et al. (24)	30	30	62±10	64±10	–	–	TXLC+CT	1 month	⑱⑳
Wang et al. (25)	44	44	57. 1 ± 7. 2	56. 5 ± 6. 3	–	–	YXSC+CT	8 weeks	①②③④⑤⑥⑦⑧
Ning et al. (26)	75	75	45–73	44 –74	4–15	4–16	YDXNTSC+CT	12 weeks	⑬⑭⑱⑲⑳㉑㉔
Zhou et al. (27)	59	59	54.2±2.6	55.3±2.1	5.1±0.2	5.2±0.5	YDXNTSC+CT	8 weeks	②⑤⑥⑧⑨⑬⑭㉒㉓㉔
Xiong et al. (28)	40	40	69.24±9.48	65.36±10.32	–	–	LWDHP+CT	2 months	②③㉕
Chen et al. (29)	32	32	63.42±4.85	64.58±4.64	4.13±1.56	4.24±1.61	SXBXP+statins+CT	8 weeks	④⑤⑥⑦⑧
Li et al. (30)	48	48	52.1±9.7	52.8±10.2	11.2±4.1	10.5±4.4	SXBXP+CT	4 weeks	①㉕
Guo (31)	31	31	64.4 ± 12.7	63.9 ± 13.1	–	–	SXBXP+trimetazidine+CT	4 months	②④⑤⑦
Wei (32)	64	64	53.4±2.1	55.1±1.9	9.1±1.2	8.9±0.7	SXBXP+CT	1 month	①
Zhang (33)	51	51	59.2±1.2	58.3±1.0	–	–	SXBXP+CT	6 months	①
Yu and Zhang (34)	55	55	–	–	–	–	YXST+CT	12 weeks	⑬
Yu (35)	55	55	–	–	–	–	YXST+CT	12 weeks	㉖
Liu (36)	41	42	64.78±7.47	64.86±7.85	–	–	YXSC+CT	6 months	②⑤⑥⑦⑧
Dong (37)	44	44	58.3±0.6	59.4±0.2	–	–	YDXNTSC+CT	1 month	①
Xing (38)	30	30	69.07±7.52	68.87±8.48	16.4±1.7	15.2±2.4	DZJTC+CT	12 weeks	②③④⑤⑥⑦⑧⑨⑩⑰
Yang et al. (39)	43	30	58.60±12.20	57.70±9.54	5.30±1.56	5.11±1.05	JLDG+CT	8 weeks	②⑳㉑
Fang and Shao (40)	60	60	60±6.8	62±6.9	5.7±1.9	5.8±1.7	SXBXP+trimetazidine	–	⑨⑩⑪
Zhang et al. (41)	50	50	59.21±1.32	59.85±1.51	–	–	SXBXP+probucol+CT	2 months	⑤⑥⑦⑧⑯⑰⑳㉑
Yang et al. (42)	62	61	61.2±7.4	60.3±5.4	–	–	TXLC+CT	12 weeks	㉕
Li (43)	42	42	61. 62 ± 6. 78	61. 74 ± 6. 89	–	–	TXLC+trimetazidine+CT	12 weeks	⑤⑥⑦⑧⑨
Bao et al. (44)	53	52	66.53±5.11	66.10±4.32	–	–	QSYQDP+CT	8 weeks	①②③④⑨⑩㉖
Zhang et al. (45)	48	48	60.21±15.37	59.87±14.65	–	–	TXLC+statins+CT	12 weeks	④⑤⑥⑦⑧
Yu et al. (46)	38	38	73.89±6.31	70.56±7.66	12.33±4.72	9.67±5.25	GXNT+CT	12 weeks	③⑤⑥⑦⑧⑰㉒㉓
Zhang (47)	50	50	66.14±2.25	66.28±2.33	–	–	TXLC+CT	–	①②⑤⑥⑧
Shi et al. (48)	60	60	80±3	79±4	–	–	YDXNTSC+CT	3 months	①⑥⑦㉖
Liang et al. (49)	51	51	73.43±3.61	73.55±3.75	–	–	SXBXP+tirofiban+CT	1 week	①⑨⑩⑮⑯㉔㉕
Zhang et al. (50)	40	40	–	–	–	–	SXBXP+CT	1 month	㉕
Zhao (52)	40	40	7 0.1 ± 9.5	7 2.5 ±1 0.4	–	–	FFDSDP+statins+CT	8 weeks	②③④⑮⑯⑰
Xie et al. (53)	75	75	75.9±12.7	75.3±12.9	12.34±5.37	12.79±7.01	YDXNTSC+CT	–	㉒㉓㉖
Li (54)	143	143	74 .84 ± 6 .47	74 .24± 6 .57	–	–	TXLC+trimetazidine+CT	3 months	②③④⑤⑥⑦⑧⑨⑩⑪
Su et al. (55)	41	41	65.3±6.1	65.5±6.2	–	–	XFZY+statins+CT	12 weeks	⑤⑥⑦⑧
Shang et al. (56)	71	71	63.68±7.37	64.26±7.02	15.26±5.25	16.31±5.67	SXBXP+tirofiban+CT	15 days	②④㉕
Mi (57)	49	49	70.36±4.55	70.51±4.83	–	–	YDXNTSC+CT	2 months	①②③⑤⑥⑬⑭㉔㉖
Jia et al. (58)	30	30	57.9±4.33	57.9±4.33	3.25±1.19	3.32±1.43	SXBXP+tirofiban+CT	2 weeks	⑨
Zhou et al. (59)	62	62	50.68±7.26	52.49±8.51	–	–	QSYQDP+CT	8 weeks	②③④⑨⑩㉖
Zhang et al. (60)	43	43	60.03±6.79	58.36±6.42	–	–	FFDSDP+CT	–	②③④⑮㉖
Liu (61)	48	48	65.02±4.30	65.34 ± 4.02	8.80±2.60	8.54± 2.65	XFZY+statins+CT	8 weeks	②③⑨⑩
Huang (62)	78	78	70.39±1.52	70.62±1.59	3.76±0.48	3.82±0.64	SXBXP+CT	3 months	②③④⑤⑥⑦⑧
Du et al. (63)	31	31	72.99±9.21	72.24±9.18	–	–	DZJTC+CT	–	⑬⑭㉖
Zhang (64)	30	30	58.12±4.06	58.04±4.10	–	–	XFZY+CT	2 months	②③④⑤⑥⑦⑧⑨⑩
Zhang et al. (65)	50	50	63.98±10.78	64.52±11.76	6.75±1.69	6.83±1.75	QSYQDP+CT	3 months	⑨⑩⑪⑫㉖
Wu et al. (66)	48	48	56.32±5.78	55.27±5.20	4.63±2.05	4.51±2.01	SXBXP+CT	2 months	②③⑤⑥⑦⑧⑮⑯㉖
Fang (67)	35	35	55.5±4.3	55.9±4.7	–	–	FFDSDP+CT	6 months	①②④⑤⑥⑧⑳㉑
Chi et al. (68)	55	55	62.56±4.84	62.73±4.45	7.93±3.57	7.83±3.82	FFDSDP+CT	4 weeks	⑨⑪
Zhou et al. (51)	340	376	64.7 ± 9.5	64.4 ± 9.3			SXBXP+CT	2 years	㉕
Zhan et al. (69)	40	56	66.8±9.9	64.2±11.2	–	–	SXBXP+CT	12 months	⑱⑲
Xi et al. (70)	90	90	65.72±9.13	66.51±7.34	–	–	FFDSDP+CT	6 months	⑱⑲
Wu et al. (71)	48	48	61.87±8.60	62.33±8.17	5.72±2.50	5.63±2.38	FRTMC+CT	4 weeks	②③④⑤⑥⑦⑳㉑㉖
Wang et al. (72)	51	51	68.55±9.67	67.90±9.51	5.96±2.02	5.80±2.11	SXBXP+probucol+CT	3 months	②③⑤⑥⑦⑧⑨⑩⑪㉑
Huang et al. (73)	53	53	65.12±7.43	66.19±6.71	5.51±1.73	5.80±1.65	GXNT+CT	3 months	②④⑨⑩㉕
Hou et al. (74)	50	50	66.71±6.61	67.24±5.57	8.79±3.11	9.38±4.78	JLDG+CT	3 months	②③④⑤⑥⑦⑧⑨⑳㉑㉖
Chen (75)	100	100	70.44±2.06	70.38±2.02	–	–	JLDG+CT	3 months	②③④⑨
Zhang (76)	32	32	54.24±4.21	53.68±4.26	4.95±0.81	4.89±0.96	SXBXP+CT	2 months	②③④⑤⑥⑦⑧⑨⑩⑪㉕
Yao et al. (77)	58	58	68.78±6.22	68.72±6.25	3.98±1.59	3.93+1.57	FRTMC+CT	1 month	①⑨⑩⑫㉕
Yang et al. (78)	78	80	64.40±9.99	64.30±8.76	–	–	SXBXP+CT	6 months	㉖
Liu (79)	25	25	70.32±5.21	70.15±5.24	–	–	SXBXP+trimetazidine	2 weeks	⑨⑩⑪
Huang et al. (80)	50	50	63.49±5.21	63.57±5.22	4.49±1.37	4.45±1.32	GXNT+CT	1 month	①②④⑤⑥⑨⑩⑯⑰㉑
Gao et al. (81)	55	55	69.09±2.08	68.97±2.23	5.49±2.21	5.51±2.09	SXBXP+probucol+CT	3 months	⑤⑥⑦⑧㉑

CT refers to Conventional Western Therapy; ① Clinical efficacy; ② FPG; ③ 2hPBG; ④ HbA1c; ⑤ TC; ⑥ TG; ⑦ LDL-C; ⑧ HDL-C; ⑨ LVEF; ⑩ LVEDD; ⑪ LVESD; ⑫ NT-proBNP; ⑬ Weekly frequency of angina pectoris episodes; ⑭ Duration of angina pectoris episodes; ⑮ IL-6; ⑯ TNF-α; ⑰ hs-CRP; ⑱ MDA; ⑲ SOD; ⑳NO; ㉑ ET-1; ㉒ Whole blood high-shear viscosity; ㉓ Whole blood low-shear viscosity; ㉔ FIB; ㉕ MACE incidence; ㉖ Adverse reactions; "–" indicates not mentioned.

The high comorbidity of CHD and DM is rooted in profound pathophysiological mechanisms. Chronic hyperglycemia, insulin resistance, and metabolic inflammation promote endothelial dysfunction, oxidative stress, macrophage activation, and unstable plaque formation, thereby accelerating the progression of atherosclerosis (4, 5). Inflammatory cytokines, including interleukin-6, tumor necrosis factor-α, and chemokine pathways, play a central role in diabetic vascular injury (6). Genetic studies have further confirmed that DM and cardiovascular diseases share several key signaling networks, encompassing multiple gene modules involved in the regulation of lipid metabolism, inflammation, and endothelial homeostasis (7).

Cardiovascular outcome studies have underscored the clinical relevance of this comorbidity. DM is recognized as a CHD risk equivalent, with the lifelong risk of atherosclerotic cardiovascular disease (ASCVD) in DM patients comparable to or even exceeding that of patients with prior myocardial infarction (8). Furthermore, accumulating evidence indicates that residual cardiovascular risk persists in DM patients even when glycemic targets are achieved, suggesting that metabolic, inflammatory, and vascular impairments cannot be fully reversed by a single therapeutic target (9). The latest Chinese expert consensus explicitly emphasizes that DM patients should be managed based on overall ASCVD risk assessment (10, 11).

Conventional Western therapies—including antiplatelet agents, lipid-lowering drugs, anti-ischemic medications, and glucose-lowering treatments—have significantly improved clinical outcomes in patients with CHD and DM. However, despite optimal control of traditional risk factors, substantial residual cardiovascular risk persists in this population. This limitation may be attributed to the predominantly single-target mechanisms of conventional therapies, which inadequately address multifactorial pathological processes such as chronic inflammation, oxidative stress, microvascular dysfunction, and metabolic dysregulation. Therefore, integrative treatment approaches with broader mechanistic coverage are increasingly being explored.

Oral Chinese patent medicines (OCPMs), characterized by multi-component and multi-target properties, have been widely used as adjunctive therapies for cardiovascular and metabolic diseases in China. Accumulating randomized controlled trials (RCTs) suggest that OCPMs combined with conventional therapy may improve glycemic control, lipid metabolism, cardiac function, angina symptoms, inflammatory responses, endothelial function, and hemorheological parameters in patients with CHD complicated by DM. Nevertheless, considerable heterogeneity exists among different OCPMs, and direct head-to-head comparisons are scarce, making it difficult to determine their relative efficacy and safety.

Network meta-analysis (NMA) enables the simultaneous comparison of multiple interventions by integrating direct and indirect evidence, thereby providing a comprehensive framework for ranking treatment options. To date, no NMA has systematically compared the efficacy and safety profiles of various OCPMs combined with conventional therapy across multiple clinically relevant outcomes in patients with CHD complicated by DM. Therefore, the present study aimed to conduct an NMA of RCTs to evaluate and rank different OCPMs in terms of glycemic control, lipid regulation, cardiac function, angina-related outcomes, inflammatory and endothelial markers, hemorheology, and safety outcomes, with the goal of providing evidence-based guidance for clinical decision-making in this high-risk comorbid population.

## 2 Methods

### 2.1 Study registration

This NMA was conducted in accordance with the guidelines of the Preferred Reporting Items for Systematic Reviews and Meta-Analyses incorporating NMA (PRISMA-NMA). The study protocol was prospectively registered on the PROSPERO platform (Registration No. CRD42024626573).

### 2.2 Literature search strategy

A comprehensive literature search was performed in the following databases: China National Knowledge Infrastructure (CNKI), VIP, WanFang Data, China Biomedical Literature Database, PubMed, Web of Science, and the Cochrane Library. RCTs evaluating OCPMs for the treatment of CHD complicated by DM were retrieved from inception to the most recent update (within the last 10 years).

**Figure 1:**
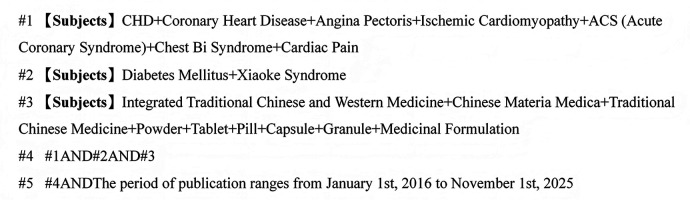
CNKI literature search strategy.

Search strategies combined controlled vocabulary terms and free-text keywords. Chinese search terms included “coronary heart disease,” “angina pectoris,” “acute coronary syndrome,” “myocardial infarction,” “diabetes,” “traditional Chinese medicine,” “oral Chinese patent medicine,” “capsule,” “tablet,” “granule,” and related synonyms. Corresponding English terms included “coronary disease,” “ischemic cardiomyopathy,” “acute coronary syndrome,” “myocardial infarction,” “diabetes mellitus,” “Chinese patent medicine,” “traditional Chinese medicine,” “capsule,” and “granule.” Taking CNKI as an example, after literature retrieval, the results were imported into NoteExpress for literature management (Figure 1).

### 2.3 Eligibility criteria

#### 2.3.1 Inclusion criteria

Studies were included if they met the following criteria: (1) RCT literature on the treatment of CHD complicated by diabetes using OCPMs. (2) The control group received conventional Western medicine treatment, including but not limited to diet and exercise intervention, lipid-lowering and plaque-stabilizing therapy, antiplatelet therapy, myocardial ischemia-improving therapy, and oral or injectable hypoglycemic drugs. The experimental group was given a single type of oral Chinese patent medicine on the basis of the control group's treatment. Except for the Chinese patent medicine, other basic treatment principles were consistent with those of the control group. (3) Outcome indicators included any one or more of the following ten categories: ① Clinical efficacy: The criteria for efficacy evaluation were formulated in accordance with the Guidelines for Clinical Trials of New Chinese Medicines. Marked effectiveness was defined as a significant improvement in angina pectoris symptoms and a return to normal heart rate as shown by electrocardiogram (ECG). Effective was defined as an elevation of the ST segment on ECG by more than 0.05 mV and a certain improvement in angina pectoris symptoms. Ineffective was defined as no improvement in ECG compared with that before treatment, with a depression of the ST segment by more than 0.05 mV. The effective rate of efficacy = (number of marked effective cases + number of effective cases)/sample size × 100%. ② Glycemic control outcomes: fasting plasma glucose (FPG), 2-h postprandial blood glucose (2 h PBG), and glycated hemoglobin (HbA1c). ③ Lipid metabolism outcomes: total cholesterol (TC), triglycerides (TG), low-density lipoprotein cholesterol (LDL-C), and high-density lipoprotein cholesterol (HDL-C). ④ Cardiac function outcomes: left ventricular ejection fraction (LVEF), left ventricular end-diastolic diameter (LVEDD), left ventricular end-systolic diameter (LVESD), and N-terminal pro-B-type natriuretic peptide (NT-proBNP). ⑤ Anti-angina efficacy outcomes: weekly frequency and duration of angina pectoris episodes. ⑥ Inflammatory response outcomes: interleukin-6 (IL-6), tumor necrosis factor-α (TNF-α), and high-sensitivity C-reactive protein (hs-CRP). ⑦ Oxidative stress outcomes: malondialdehyde (MDA) and superoxide dismutase (SOD). ⑧ Endothelial function outcomes: nitric oxide (NO) and endothelin-1 (ET-1). ⑨ Hemorheology outcomes: whole blood high-shear viscosity, whole blood low-shear viscosity, and fibrinogen (FIB). ⑩ Safety outcomes: MACE and adverse reactions.

#### 2.3.2 Exclusion criteria

Studies were excluded if they met any of the following conditions: (1) duplicate publications or reports with overlapping data; (2) lacked full text or sufficient outcome data; (3) non-clinical studies (e.g., animal or cell experiments); (4) used non-randomized designs or lacked a control group; (5) employed more than one OCPM or combined OCPMs with other traditional Chinese medicine interventions such as acupuncture or decoctions; (6) did not report any predefined outcome of interest; and (7) reviews, meta-analyses, conference abstracts, or other non-original studies.

### 2.4 Study selection and data extraction

Two investigators independently screened titles and abstracts, followed by full-text assessment according to the eligibility criteria. Disagreements were resolved through discussion with a third investigator. Data were extracted using a standardized data collection form, including study characteristics (author, publication year, sample size), patient demographics, intervention details (type of OCPM, treatment duration), and reported outcomes. For studies reporting outcomes at multiple time points, data at the end of treatment were preferentially extracted.

### 2.5 Risk of bias assessment

The methodological quality of included studies was assessed using the Cochrane Risk of Bias Tool, evaluating random sequence generation, allocation concealment, blinding of participants and outcome assessors, completeness of outcome data, selective reporting, and other potential sources of bias. Two reviewers independently performed the assessment, with discrepancies resolved by consensus.

### 2.6 Statistical analysis

The NMA was conducted using R (version 4.5.2) and Stata (version 18.0). Odds ratios (ORs) were calculated for dichotomous outcomes, while mean differences were used for continuous outcomes, both with 95% confidence intervals (CIs). Treatment ranking was assessed using the surface under the cumulative ranking curve (SUCRA). Model convergence was evaluated using the potential scale reduction factor (PSRF), with values <1.1 indicating adequate convergence. Sensitivity analyses were performed by sequentially excluding individual studies. Publication bias was assessed using comparison-adjusted funnel plots and Egger's test.

## 3 Results

### 3.1 Study selection

**Figure 2:**
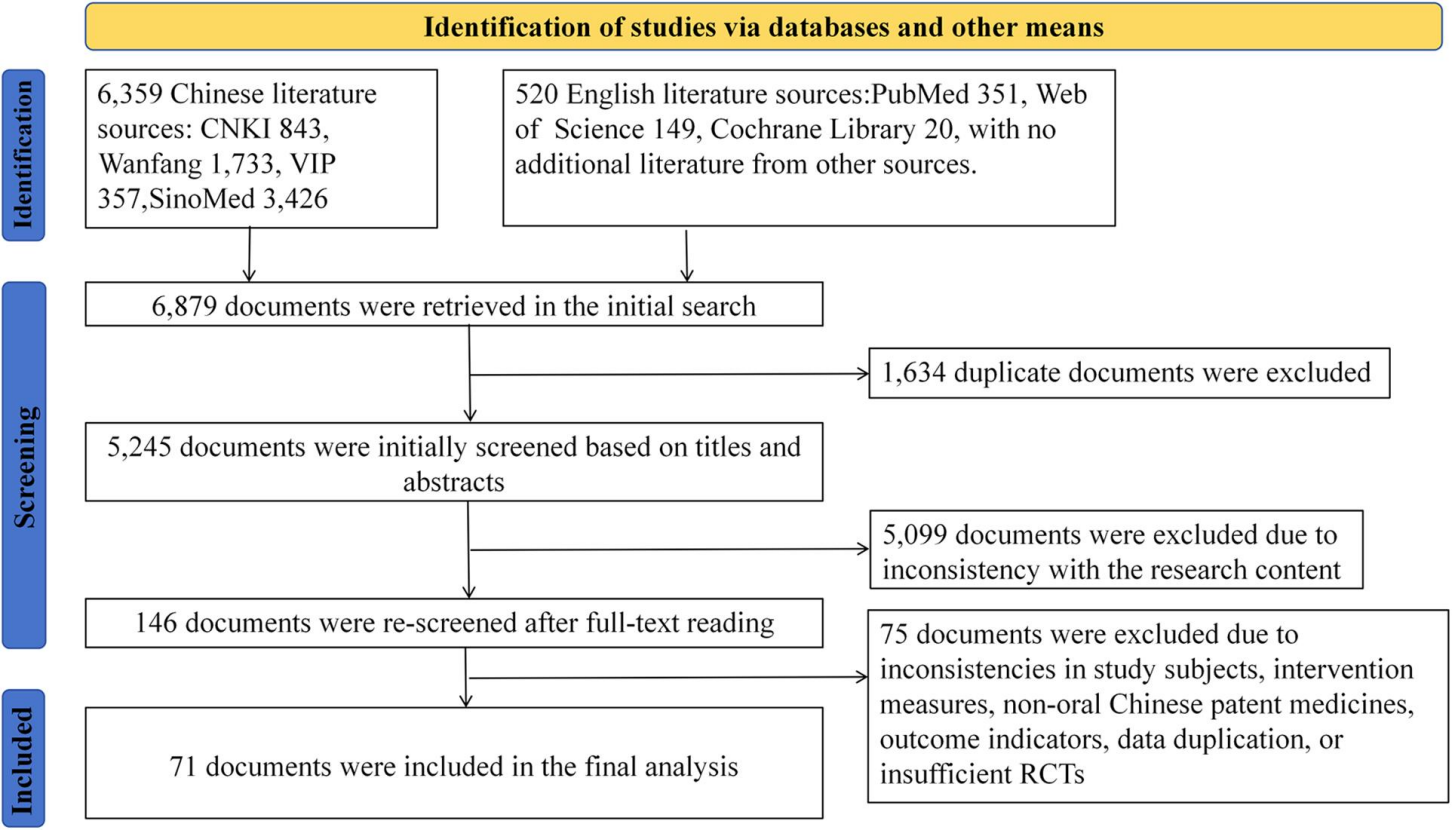
Literature screening process.

We retrieved a total of 6,879 articles from the above-mentioned databases based on the search strategy, including 6,359 Chinese articles and 520 foreign-language articles. We excluded 6,789 articles according to the inclusion and exclusion criteria, and finally included 71 articles, with 70 in Chinese and one in English (Figure 2).

### 3.2 Study characteristics

A total of 71 (12–80) studies were included, all of which were two-arm trials, involving 7,988 patients, with 3,975 in the experimental group and 4,013 in the control group. Fourteen OCPMs were investigated: Shexiang Baoxin Pill (SXBXP), Tongxinluo Capsule (TXLC), Fufang Danshen Dripping Pill (FFDSDP), Yindan Xinnaotong Soft Capsule (YDXNTSC), Qishen Yiqi Dripping Pill (QSYQDP), Guanxinning Tablet (GXNT), Jinlida Granule (JLDG), Liuwei Dihuang Pill (LWDHP), Yangxinshi Tablet (YXST), Xuefu Zhuyu Pill/Tablet (XFZY), Danzhi Jiangtang Capsule (DZJTC), Furong Tongmai Capsule (FRTMC), Guanxin Xiaokean Capsule (GXXKAC), and Yixinshu Capsule (YXSC).

### 3.3 Risk of bias

**Figure 3:**
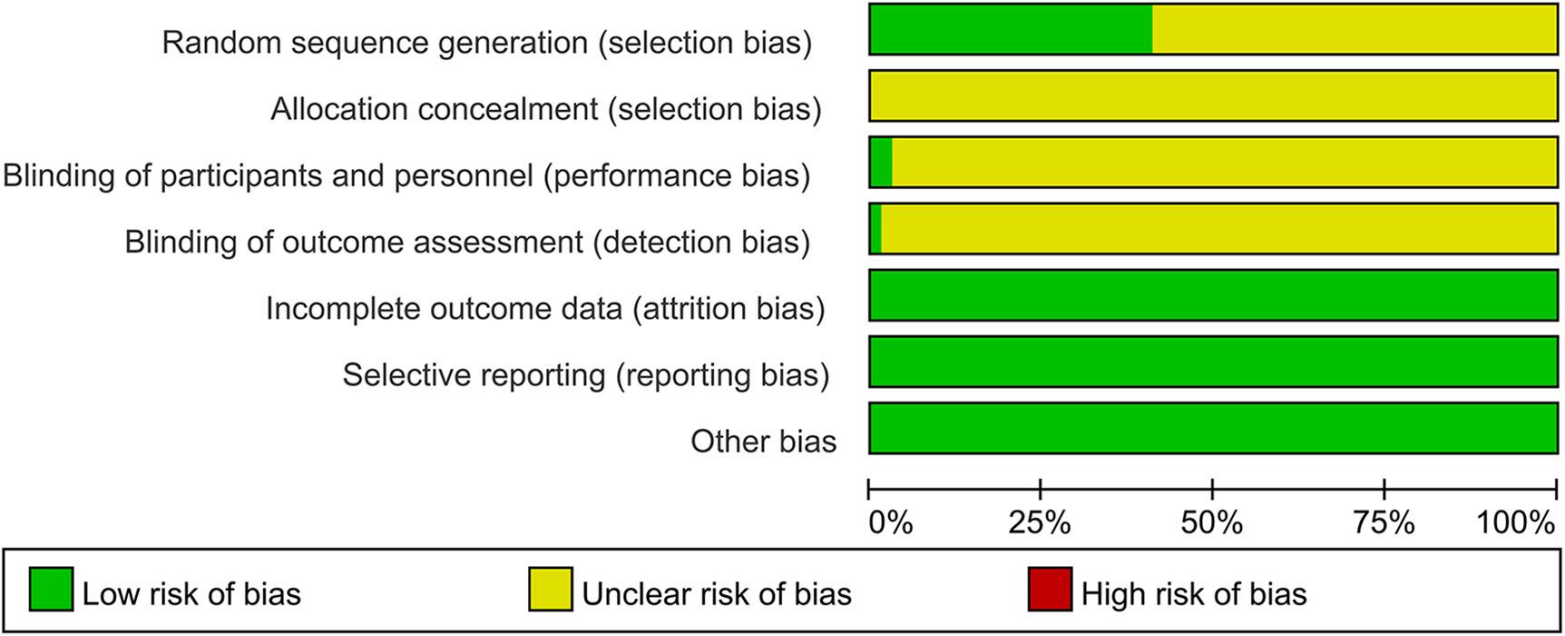
Cochrane risk of bias assessment for included studies.

The Risk of Bias Assessment Tool in Review Manager 5.4 was used to evaluate the included studies. A total of 29 studies (14, 15, 19, 31, 35, 38, 39, 44, 49, 56–62, 64, 65, 67, 70–72, 74, 75, 77–79) adopted the correct randomization method and were judged to be “Low risk,” whereas the remaining studies did not describe the specific randomization protocol and were judged to be “Unclear risk.” None of the protocols described allocation concealment, and all were judged to be “Unclear risk.” One study (68) adopted a “double-blind” design and was judged to be “Low risk,” and one study (15) implemented blinding of researchers and was also judged to be “Low risk.” However, the remaining studies did not specifically describe blinding methods and were thus judged to be “Unclear risk.” All studies reported complete data without case loss and were judged to be “Low risk.” All included studies reported all observation indicators and were judged to be “Low risk.” No other biases were found in the studies, which were judged to be “Low risk” (Figure 3, Supplementary Figure S1).

### 3.4 Evidence network

**Figure 4:**
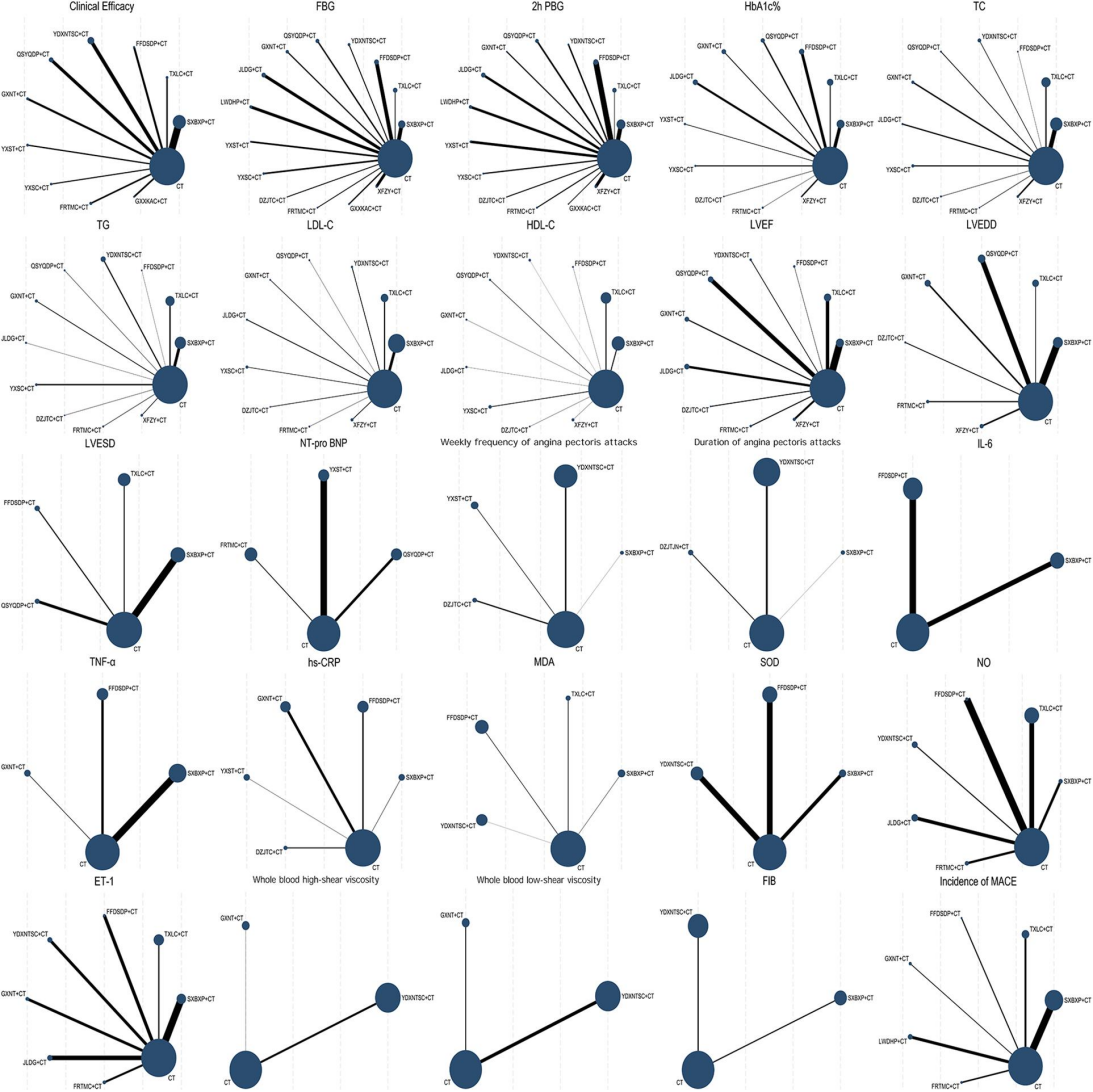
Evidence network diagrams of each outcome indicator.

Evidence network diagrams were plotted for all outcome indicators of 19 different OCPMs used in the treatment of CHD complicated by DM (Figure 4). In the evidence network, the dots represent different interventions, with the size of the dots indicating the sample size of patients—the larger the dot, the larger the sample size of patients included in the treatment. The thickness of the connecting lines represents the number of included comparative studies—the thicker the line, the greater the number of studies.

#### 3.5.1 Inconsistency test

As shown in Figure 4, no closed loops were formed in the evidence network for the outcome indicators in this study, and thus an inconsistency test was not required.

#### 3.5.2 Evaluation of iterative convergence

Based on the Markov chain Monte Carlo algorithm, the iterative convergence evaluation of each outcome indicator demonstrated that all PSRF values were less than 1.1. This indicated good model convergence and high credibility of the analysis results.

#### 3.5.3 Evaluation of leverage–residual deviance

The diagnostic results of the NMA model for each outcome indicator revealed that the residuals of all indicators mostly fell within the reasonable range of 0–1.5, suggesting excellent model fitting, with local fluctuations providing references for future research. The data were dominated by the pattern of “low leverage–low residual,” indicating that the core data were stable and verifying the reliability of the results (Supplementary Figure S2).

### 3.6 Clinical efficacy

**Figure 5:**
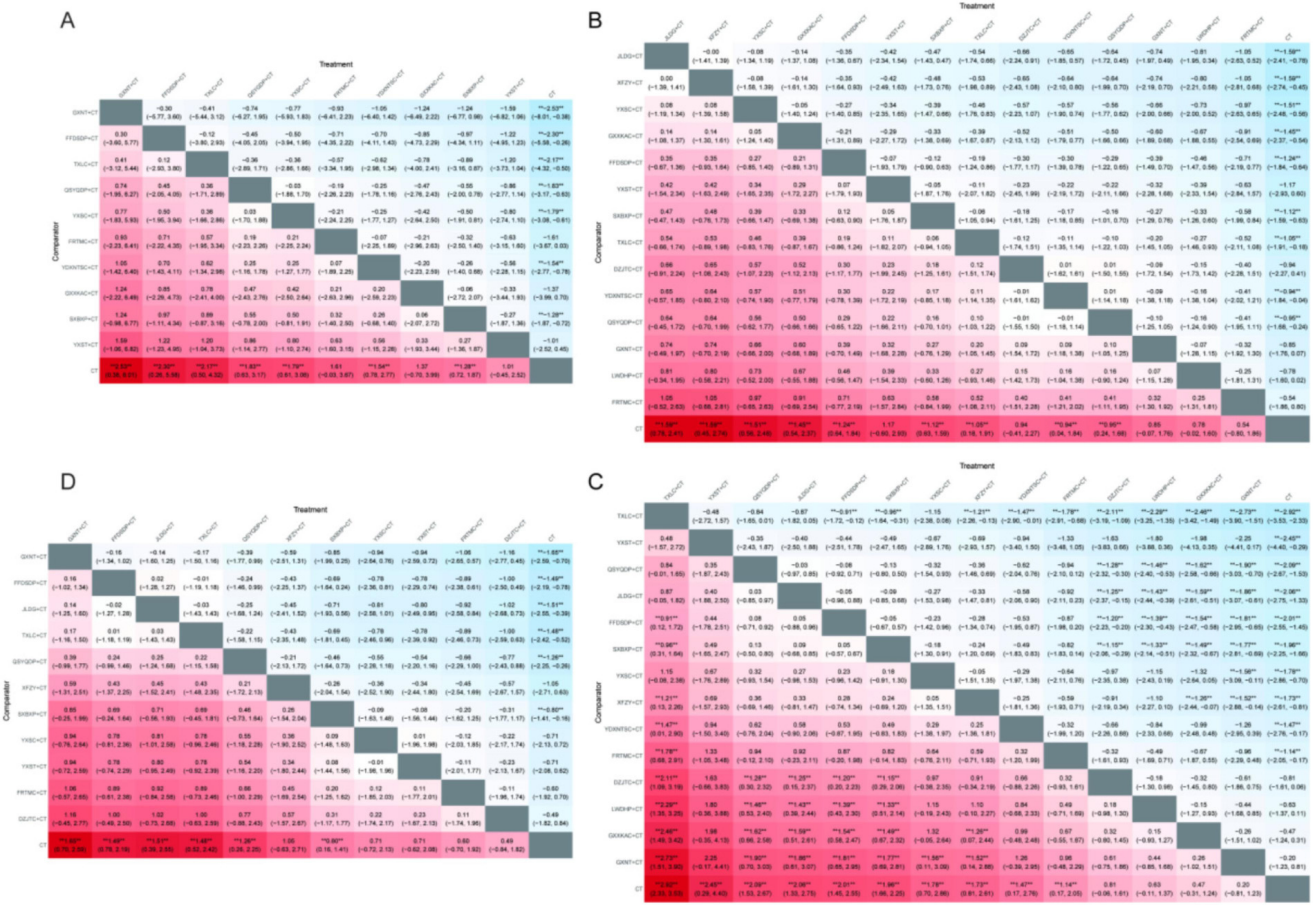
Heatmaps of clinical efficacy and glycemic control outcomes. **(A)** Clinical efficacy; **(B)** FBG; **(C)** 2 h PBG; **(D)** HbA1c. ** indicates a statistically significant difference between the two interventions (*P* < 0.05).

A total of 18 studies (13, 17, 21, 22, 24, 26, 30, 32, 33, 37, 44, 47–49, 56, 66, 76, 79) involving 10 OCPMs and 1,757 patients reported clinical efficacy outcomes. The NMA showed that most combination therapies were more effective than CT, while no statistically significant differences were observed among the interventions (Figure 5A).

### 3.7 Glycemic control outcomes

#### 3.7.1 FPG

A total of 36 studies (12–14, 17–21, 23, 26, 28, 31, 36, 38, 39, 44, 47, 51, 53, 55, 56, 58–61, 63, 65, 66, 70–75, 79) involving 14 OCPMs and 3,572 patients reported FPG outcomes. The NMA showed that, compared with conventional CT, combination therapies were generally more effective. In particular, JLDG + CT, XFZY + CT, and YXSC + CT demonstrated significantly improved efficacy. No statistically significant differences were observed among most of the combination therapies (Figure 5B).

#### 3.7.2 2-h PBG

A total of 26 studies (23, 24, 26, 28, 38, 44, 46, 51, 53, 56, 58–61, 63, 65, 70, 71, 73–75) involving 14 OCPMs and 2,638 patients reported 2-h PBG outcomes. The NMA showed that most combination therapies were more effective than CT. Among them, TXLC + CT demonstrated superior efficacy in most comparisons, whereas DZJTC + CT, LWDHP + CT, GXXKAC + CT, and GXNT + CT showed relatively poorer performance; some comparisons reached statistical significance (Figure 5C).

#### 3.7.3 Hba1c

A total of 22 studies (17, 19, 26, 29, 31, 38, 44, 45, 51, 53, 55, 58, 59, 61, 63, 66, 70, 72–75, 79) involving 11 OCPMs and 2,319 patients reported HbA1c outcomes. The NMA showed that all combination therapies were more effective than CT, with some comparisons reaching statistical significance. However, differences among the combination therapies were generally small, and most comparisons were not statistically significant (Figure 5D).

### 3.8 Lipid metabolism outcomes

#### 3.8.1 TC

**Figure 6:**
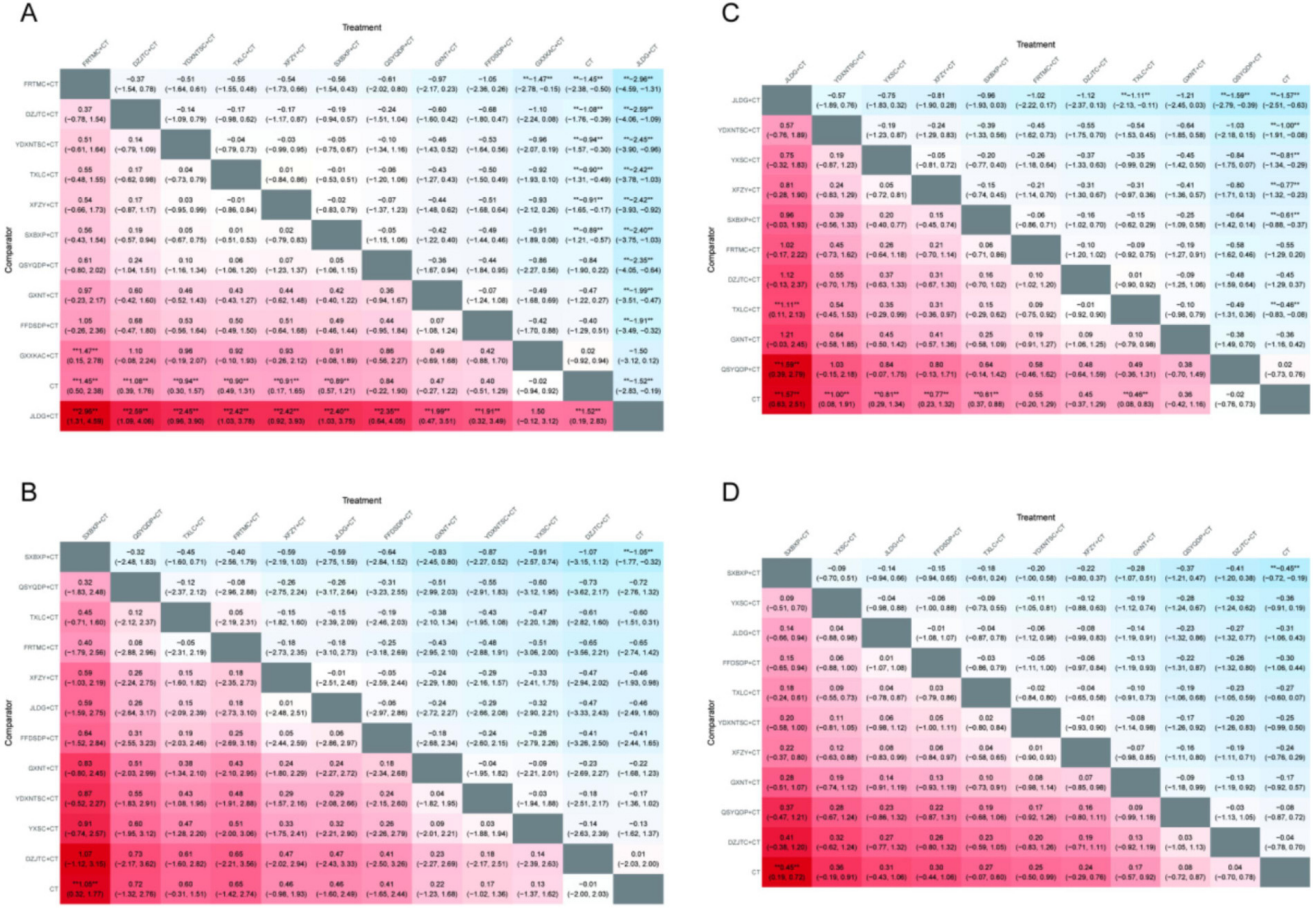
Heatmaps of lipid metabolism outcomes. **(A)** TC; **(B)** TG; **(C)** LDL-C; **(D)** HDL-C. ** indicates a statistically significant difference between the two interventions (*P* < 0.05).

A total of 27 studies (16, 19, 21, 22, 26, 29, 31, 36, 38, 41, 43, 45–47, 53, 54, 56, 61, 63, 65, 66, 70, 71, 73, 75, 79, 80) involving 11 OCPMs and 2,531 patients reported TC outcomes. The NMA showed that, compared with CT, combination therapies were generally more effective, with some comparisons reaching statistical significance. In addition, most interventions demonstrated superior efficacy compared with JLDG + CT, while no statistically significant differences were observed among most combination therapies (Figure 6A).

#### 3.8.2 TG

A total of 27 studies (16, 19, 21, 22, 26, 29, 36, 38, 41, 43, 45–48, 53, 54, 56, 61, 63, 65, 66, 70, 71, 73, 75, 79, 80) involving 11 OCPMs and 2,589 patients reported TG outcomes. The NMA showed that most interventions did not differ significantly from CT, with only SXBXP + CT showing a statistically significant difference. In addition, differences among the combination therapies were small, and most comparisons were not statistically significant (Figure 6B).

#### 3.8.3 LDL-C

A total of 23 studies (16, 21, 22, 26, 29, 31, 36, 38, 41, 43, 45, 46, 48, 53, 54, 61, 63, 65, 70, 71, 73, 75, 80) including 10 OCPMs and 2,165 patients reported LDL-C outcomes. The NMA showed that most combination therapies were more effective than CT. Notably, JLDG + CT demonstrated superior efficacy compared with TXLC + CT and QSYQDP + CT in some comparisons, while most differences among the combination therapies were not statistically significant (Figure 6C).

#### 3.8.4 HDL-C

A total of 23 studies (16, 19, 21, 22, 26, 29, 36, 38, 41, 43, 45–47, 53, 54, 61, 63, 65, 66, 71, 73, 75, 80) involving 10 OCPMs and 2,175 patients reported HDL-C outcomes. The NMA showed that only SXBXP + CT showed a statistically significant difference compared with CT, while most other comparisons were not statistically significant (Figure 6D).

### 3.9 Cardiac function outcomes

#### 3.9.1 LVEF

**Figure 7:**
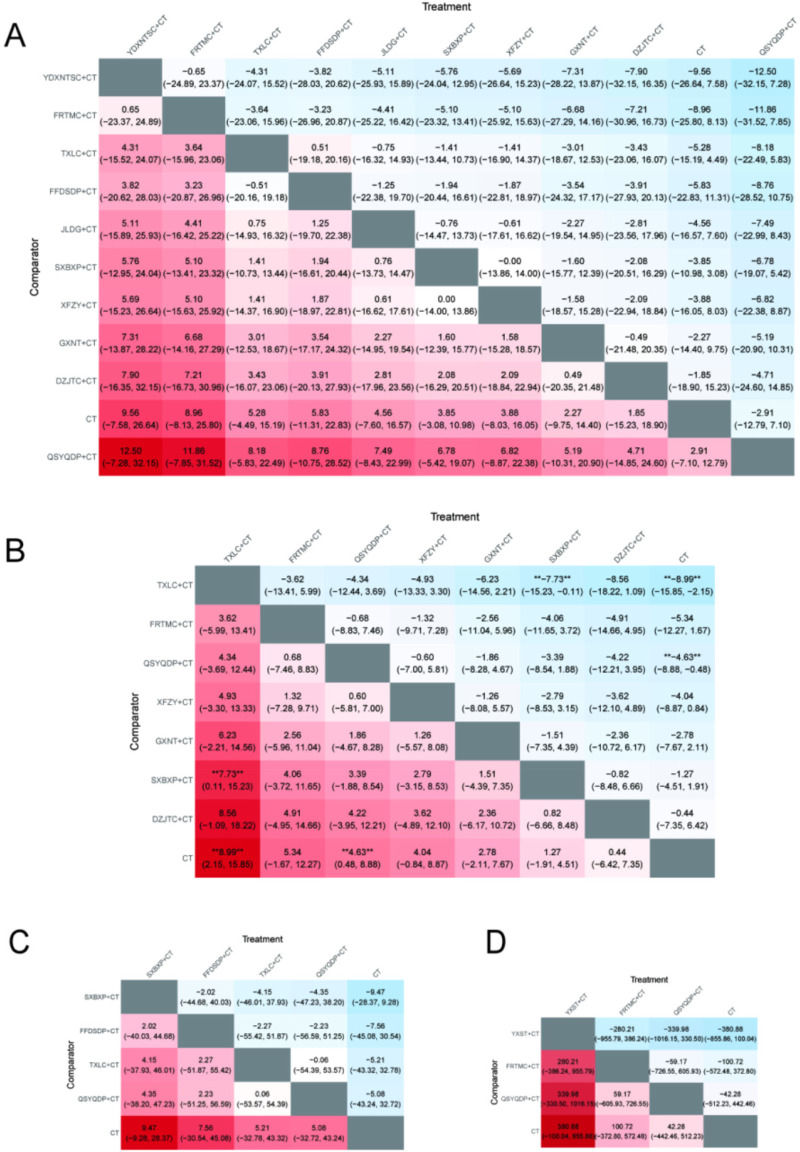
Heatmaps of cardiac function outcomes. **(A)** LVEF; **(B)** LVEDD; **(C)** LVESD; **(D)** NT-pro-BNP. ** indicates a statistically significant difference between the two interventions (*P* < 0.05).

A total of 22 studies (16, 19, 38, 40, 43, 44, 49, 53, 57, 58, 60, 63, 64, 67, 71–76, 78, 79) reported LVEF outcomes, involving 10 OCPMs and a total of 2,343 enrolled patients. The NMA showed that no statistically significant differences were observed among the interventions (Figure 7A).

#### 3.9.2 LVEDD

A total of 15 studies (38, 40, 44, 49, 53, 58, 60, 63, 64, 71, 72, 75, 76, 78, 79) reported LVEDD outcomes, involving seven OCPMs and a total of 1,591 enrolled patients. The NMA showed that some combination therapies were more effective than CT. Among them, TXLC + CT, DZJTC + CT, and QSYQDP + CT showed statistically significant differences in some comparisons, while most differences among the combination therapies were not statistically significant (Figure 7B).

#### 3.9.3 LVESD

Seven studies (40, 53, 64, 67, 71, 75, 78) reported LVESD outcomes, involving four OCPMs and a total of 832 enrolled patients. No statistically significant differences were observed among the interventions (Figure 7C).

#### 3.9.4 NT-proBNP

Three studies (17, 64, 76) reported NT-proBNP outcomes, involving three OCPMs and a total of 316 enrolled patients. No statistically significant differences were observed among the interventions (Figure 7D).

### 3.10 Angina-related outcomes

#### 3.10.1 Weekly angina attack frequency

**Figure 8:**
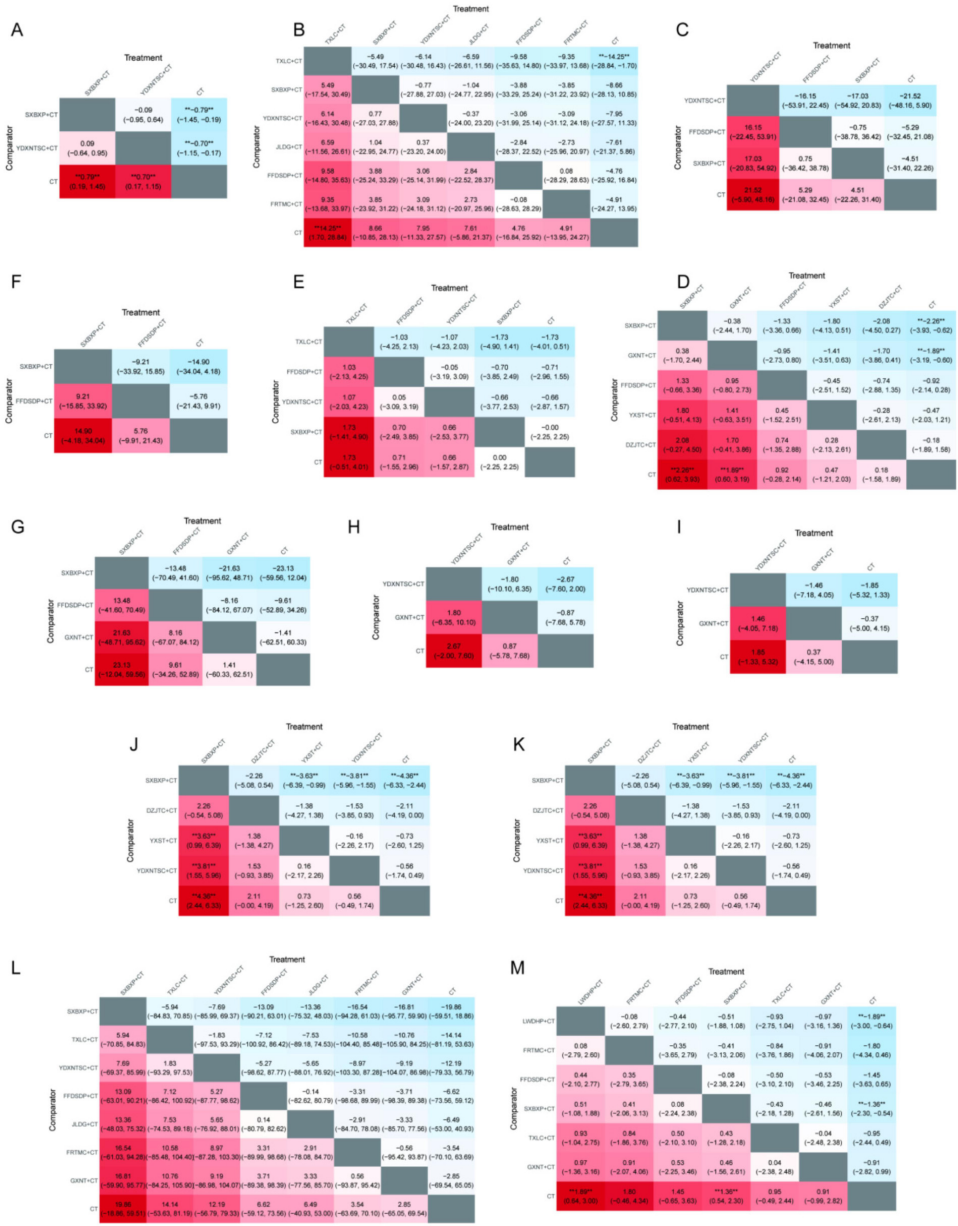
Heatmaps of others. **(A)** FIB; **(B)** NO; **(C)** SOD; **(D)** hs-CRP; **(E)** MDA, **(F)** IL-6; **(G)** TNF-α; **(H)** whole blood viscosity at low shear rate; **(I)** whole blood viscosity at high shear rate; **(J)** weekly angina pectoris attack frequency; **(K)** angina pectoris duration; **(L)** ET-1; **(M)** MACE. ** indicates a statistically significant difference between the two interventions (*P* < 0.05).

Six studies (19, 23, 27, 34, 56, 62) involving four OCPMs and 586 patients reported weekly angina pectoris attack frequency. The NMA showed that SXBXP + CT demonstrated superior efficacy compared with other interventions, with some comparisons reaching statistical significance. Most differences among the remaining interventions were not statistically significant (Figure 8J).

#### 3.10.2 Duration of angina attacks

Five studies (19, 23, 27, 56, 62) involving three OCPMs and 476 patients reported the duration of angina pectoris attacks. No statistically significant differences were observed among the interventions (Figure 8K).

### 3.11 Inflammatory outcomes

Five studies (18, 49, 51, 59, 65) involving two OCPMs and 473 patients reported IL-6 outcomes, while six studies (18, 41, 49, 51, 65, 79) involving three OCPMs and 587 patients reported TNF-α outcomes (Figures 8F,G). No statistically significant differences were observed among the interventions. Seven studies (17, 18, 38, 41, 46, 51, 79) involving five types of OCPMs and 625 patients reported hs-CRP outcomes. The NMA showed that SXBXP + CT and GXNT + CT were more effective than CT, while most differences among the remaining interventions were not statistically significant (Figure 8D).

### 3.12 Oxidative stress outcomes

Four studies (25, 27, 50, 69) involving four OCPMs and 486 patients reported MDA outcomes, and three studies (27, 50, 69) involving three OCPMs and 410 patients reported SOD outcomes. No statistically significant differences were observed among the interventions (Figures 8C,E).

### 3.13 Endothelial function outcomes

#### 3.13.1 NO

Eight studies (15, 25, 27, 39, 41, 66, 70, 73) involving six OCPMs and 992 patients reported NO outcomes. The NMA showed that most interventions did not differ significantly from CT, with only TXLC + CT demonstrating superior efficacy compared with CT. Most differences among the remaining interventions were not statistically significant (Figure 8B).

#### 3.13.2 ET-1

A total of 10 studies (15, 27, 39, 41, 66, 70, 71, 73, 79, 80) involving seven OCPMs and 1,134 patients reported ET-1 outcomes. No statistically significant differences were observed among the interventions (Figure 8L).

### 3.14 Hemorheological outcomes

#### 3.14.1 High-shear whole blood viscosity and low-shear whole blood viscosity

Three studies (19, 46, 52) involving two OCPMs and 344 patients reported high-shear and low-shear whole blood viscosity outcomes. No statistically significant differences were observed among interventions (Figure 8H,I).

#### 3.14.2 FIB

Five studies (19, 22, 27, 49, 56) involving two OCPMs and 588 patients reported fibrinogen outcomes. The NMA showed that combination therapies were more effective than CT, while no statistically significant differences were observed among the combination therapies (Figure 8A).

### 3.15 Safety outcomes

#### 3.15.1 MACE

A total of 14 studies (14, 15, 19, 20, 28, 30, 42, 49, 50, 55, 68, 72, 75, 76) involving six OCPMs and 2,242 patients reported MACE outcomes. The NMA showed that LWDHP + CT and SXBXP + CT were more effective than CT, while the other interventions did not show statistically significant differences compared with CT (Figure 8M).

#### 3.15.2 Adverse reactions

A total of 14 studies reported adverse reactions, which mainly involved the circulatory system, digestive system, endocrine system, as well as skin rashes and epistaxis (Table 2).

**Table 2:** Incidence of adverse reactions.

Study	Adverse reactions	
	Experimental group	Control group
Li (16)	Diarrhea, 1 case	Palpitations, 2 cases; fatigue, 1 case; diarrhea, 1 case; headache, 1 case; dry mouth, 2 cases; gastrointestinal discomfort, 2 cases
Yu and Zhang (33)	None	None
Bao et al. (43)	Nausea and vomiting, 1 case; diarrhea, 3 cases; transient hypoglycemia, 1 case	Nausea and vomiting, 1 case; diarrhea, 3 cases; transient hypoglycemia, 1 case
Shi et al. (47)	Two patients experienced acid reflux and abdominal distension due to medication administration before meals; the symptoms of acid reflux and discomfort resolved after medication was taken postprandially.	None
Xie et al. (52)	Nausea and vomiting, 1 case	Nausea and vomiting, 2 cases; diarrhea, 2 cases
Mi (56)	Abdominal distension, 1 case; acid reflux, 1 case; epistaxis, 1 case	Abdominal distension, 2 cases; acid reflux, 1 case; epistaxis, 1 case; dyspnea, 1 case
Zhou et al. (58)	Nausea, 1 case; diarrhea, 1 case; constipation, 1 case	Nausea, 1 case; abdominal pain, 1 case; constipation, 2 cases
Zhang et al. (59)	Gastrointestinal reaction, 1 case; nausea, 1 case	Gastrointestinal reaction, 1 case
Du et al. (62)	None	None
Zhang et al. (64)	None	None
Wu and Liu (65)	Skin rash, 1 case; headache, 1 case	Skin rash, 2 cases; hypotension, 1 case; headache, 3 cases
Wu et al. (70)	Nausea, 1 case; diarrhea, 1 case	Dizziness, 1 case; nausea, 2 cases; diarrhea, 2 cases
Hou et al. (73)	Hypoglycemia, 5 cases; gastrointestinal discomfort, 4 cases; skin rash, 1 case; dizziness and headache, 2 cases	Hypoglycemia, 6 cases; gastrointestinal discomfort, 3 cases; dizziness and headache, 1 case
Yang et al. (77)	Abdominal distension, nausea and anorexia, 11 cases in total	Abdominal distension, nausea and anorexia, 10 cases in total

### 3.16 SUCRA ranking

**Figure 9:**
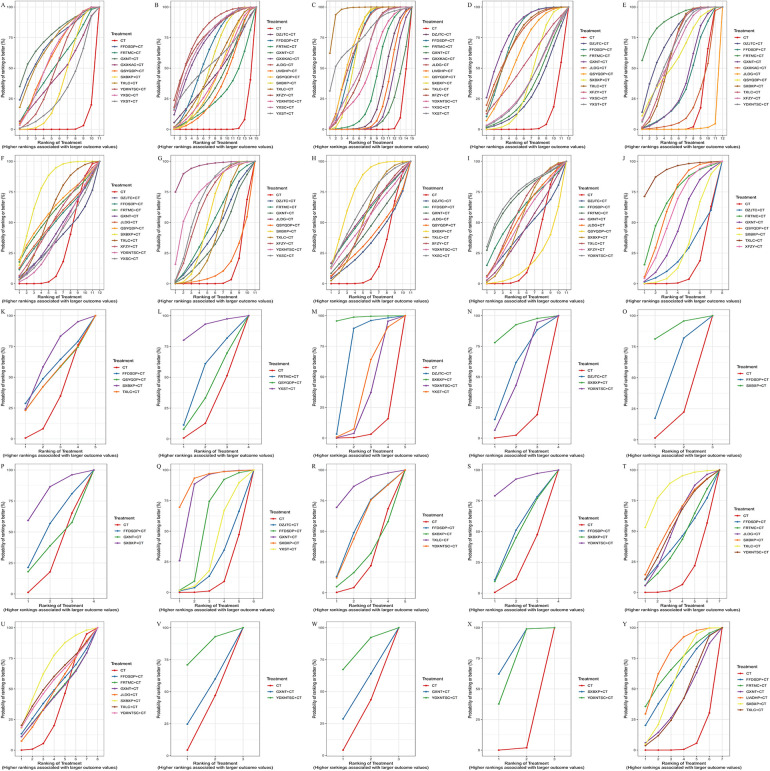
Cumulative probability ranking of oral Chinese patent medicines for the treatment of CHD complicated by DM. **(A)** Clinical efficacy; **(B)** FPG; **(C)** 2 h PBG; **(D)** HbA1c; **(E)** TC; **(F)** represents TG; **(G)** LDL; **(H)** HDL; **(I)** LVEF; **(J)** LVEDD; **(K)** LVESD; **(L)** NT-proBNP; **(M)** the number of weekly angina pectoris attacks; **(N)** the duration of angina pectoris; **(O)** IL-6; **(P)** TNF-α; **(Q)** hs-CRP; **(R)** MDA; **(S)** SOD; **(T)** NO; **(U)** ET-1; **(V)** whole blood high-shear viscosity; **(W)** whole blood low-shear viscosity; **(X)** FIB; **(Y)** the incidence of MACE.

The SUCRA ranking of different Chinese patent medicines combined with conventional Western medicine showed the following results: SXBXP combined with conventional Western medicine exhibited optimal efficacy in TG, improving HDL-C and LVESD, decreasing the weekly frequency and duration of angina pectoris episodes, and lowering levels of IL-6, TNF-α, hs-CRP, ET-1, and FIB. TXLC combined with conventional Western medicine achieved the best outcomes in clinical efficacy, reducing 2-h PBG, improving LVEDD, increasing NO, and improving MDA. YDXNTSC combined with conventional Western medicine showed the most prominent effects in enhancing LVEF, reducing whole blood high-shear viscosity and low-shear viscosity, and improving SOD. GXNT combined with conventional Western medicine demonstrated optimal efficacy in lowering HbA1c. JLDG combined with conventional Western medicine yielded the best results in reducing fasting blood glucose (FBG) and LDL-C. LWDHP combined with conventional Western medicine presented optimal efficacy in reducing the incidence of MACE. YXST combined with conventional Western medicine exhibited the most significant effect in decreasing NT-proBNP. DZJTC combined with conventional Western medicine achieved the best outcomes in reducing TC (Figure 9, Supplementary Table S1).

### 3.17 Assessment of publication bias

**Figure 10:**
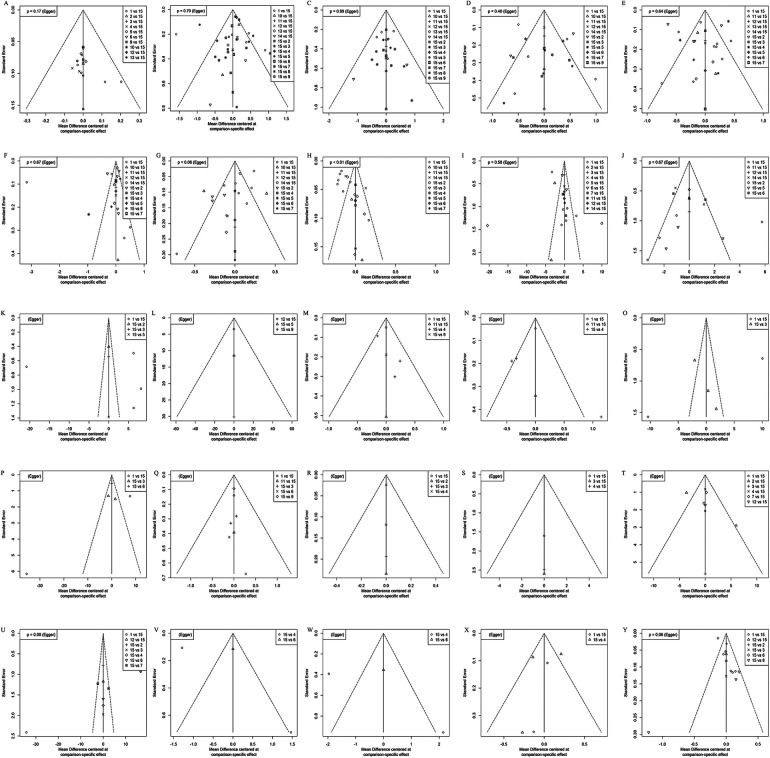
Calibrated funnel plots of various outcome indicators for OCPMs in the treatment of CHD complicated by DM. **(A)** Clinical efficacy; **(B)** FPG; **(C)** 2 h PBG; **(D)** HbA1c; **(E)** TC; **(F)** represents TG; **(G)** LDL; **(H)** HDL; **(I)** LVEF; **(J)** LVEDD; **(K)** LVESD; **(L)** NT-proBNP; **(M)** the number of weekly angina pectoris attacks; **(N)** the duration of angina pectoris; **(O)** IL-6; **(P)** TNF-α; **(Q)** hs-CRP; **(R)** MDA; **(S)** SOD; **(T)** NO; **(U)** ET-1; **(V)** whole blood high-shear viscosity; **(W)** whole blood low-shear viscosity; **(X)** FIB; **(Y)** the incidence of MACE.

Corrected funnel plots were drawn for each outcome indicator, and Egger's test was performed. The results showed that the funnel plots had poor left–right symmetry, with some points scattered outside the 95% CI region. Egger's test yielded *P* < 0.05, suggesting that the included studies may have a certain degree of publication bias or small-sample effect (Figure 10).

### 3.18 CINeMA (confidence in network meta-analysis) evidence quality assessment

This study employed the CINeMA online tool to conduct a graded assessment of the evidence quality of network meta-analysis results (81, 82). The evaluation dimensions cover six major aspects—within-study bias, between-study bias, indirectness, imprecision, heterogeneity, and inconsistency—with the evidence quality classified into four grades—high, moderate, low, and very low. After assessment, the evidence quality of the comparative results of the included interventions was mostly concentrated in the low and very low grades. The main downgrading factors were as follows: (1) Most included studies did not elaborate on the random sequence generation method, nor did they clearly report the allocation concealment scheme and blinding implementation details, resulting in a high risk of research bias. (2) There were differences among the included studies in terms of disease diagnostic criteria, efficacy evaluation standards, intervention courses, sample sizes, and TCM syndrome differentiation criteria. In particular, some studies failed to clearly specify the TCM syndromes in patients with CHD complicated with DM. In addition, the standards used for syndrome classification and differentiation were inconsistent across studies. These issues significantly reduced the consistency and accuracy of the pooled results. (3) There were varying degrees of clinical, statistical, and TCM syndrome-related heterogeneity among the studies, and the imbalance in syndrome distribution further reduced the overall evidence quality. (4) There were differences in the administration regimens of OCPMs. In some studies, the dosage and frequency of the same oral Chinese patent medicine were not completely unified, and the treatment duration varied greatly (from 1 to 2 weeks to up to 2 years). The inconsistency in administration regimens exacerbated the heterogeneity of the results. (5) There were differences in the details of basic treatment. Although all studies were based on conventional Western medicine treatment, the specific selection and usage of hypoglycemic drugs, lipid-lowering drugs, and antiplatelet drugs varied among different studies, introducing potential confounding factors. (6) There was heterogeneity in the measurement of outcome indicators. The specific judgment details of some clinical efficacy evaluation standards and the detection methods and reagents of laboratory indicators were inconsistent, which may have led to result bias. ⑦ The baseline characteristics of patients were not fully matched. Some studies did not report key characteristics in detail, such as comorbidities, smoking and drinking history, and BMI. These factors may have affected drug response and increased heterogeneity. (8) There were obvious regional and population differences. Most of the included studies were single-center studies in China, and there were potential differences in medical levels and patients' living habits among different regions, leading to regional heterogeneity in treatment effects (Supplementary Table S2).

## 4 Discussion

This study employed an NMA, incorporating 71 RCTs to systematically compare the efficacy of 14 OCPMs combined with conventional Western medicine in the treatment of CHD complicated by DM. Comprehensive evaluations were conducted across multiple dimensions, including blood glucose, blood lipids, cardiac function, angina pectoris, oxidative stress, inflammation, endothelial function, and hemorheology, aiming to provide evidence-based support for clinical medication selection.

CHD complicated by DM is increasingly recognized as a metabolic–vascular–myocardial continuum driven primarily by insulin resistance (83–85). Against this pathological backdrop, metabolic disturbances, oxidative stress, endothelial dysfunction, microcirculatory perfusion deficits, and alterations in myocardial structure and function do not occur in isolation but evolve synergistically along a highly interconnected and mutually reinforcing pathological axis (86–88). Therefore, the evaluation of therapeutic efficacy in this disease should be based on integrated improvements across multidimensional outcome measures, rather than relying on a single clinical or biochemical endpoint (89–91). In line with this perspective, traditional Chinese medicine theory likewise emphasizes holistic regulation, attributing the pathogenesis of this condition primarily to congenital insufficiency, dietary irregularities, emotional disturbances, and internal injury due to overexertion. As the disease progresses, it often manifests as a pattern of dual deficiency of both *qi* and *yin* combined with phlegm–stasis intermingling, forming a complex pattern of root deficiency and branch excess (92). Based on this understanding, OCPMs—characterized by their multi-pathway, multi-target therapeutic effects, as well as advantages such as diverse formulations and ease of storage and transport—have gained broad acceptance among both physicians and patients in clinical practice (93). Various OCPMs have been used in the clinical management of this disease and have demonstrated favorable therapeutic outcomes. However, the comparative efficacy among these different medicines remains unclear, underscoring the urgent need for systematic evaluation to differentiate them.

### 4.1 Ranking of efficacy advantages and quality of evidence for different OCPMs

The NMA results showed that, in most cases, OCPMs combined with conventional Western therapy were more effective than conventional Western therapy alone. According to the SUCRA rankings of different OCPMs, SXBXP combined with CT showed the best efficacy in reducing triglycerides, improving LVESD, reducing the weekly frequency and duration of angina attacks, and decreasing IL-6, TNF-α, hs-CRP, ET-1, and FIB. TXLC combined with CT showed the best overall clinical efficacy, reducing 2-h PBG, improving LVEDD, increasing NO, and improving MDA. YDXNTSC combined with CT showed the best efficacy in increasing LVEF, reducing whole blood high-shear and low-shear viscosity, and improving SOD. GXNT combined with CT showed the best efficacy in reducing glycated hemoglobin HbA1c. JLDG combined with CT showed the best efficacy in reducing FBG and LDL-C. LWDHP combined with CT showed the best efficacy in reducing the incidence of MACE. YXST combined with CT showed the best efficacy in reducing NT-proBNP. Finally, DZJTC combined with CT showed the best efficacy in reducing TC.

The results of the CINeMA evidence quality assessment revealed variability across these efficacy indicators, with most being rated as low or very low quality. Limitations related to the risk of bias, imprecision, and heterogeneity were among the primary factors. Therefore, interpretation of the ranking results should be approached with caution, taking into account the evidence grades.

### 4.2 Preclinical mechanistic evidence

Preclinical studies have provided substantial mechanistic evidence supporting the cardiovascular protective effects of several OCPMs in the context of CHD complicated by DM.

#### 4.2.1 SXBXP: anti-inflammatory, antioxidant, and pro-angiogenic effects

*In vivo* studies have demonstrated that SXBXP can downregulate pro-inflammatory cytokines and adhesion molecules (TNF-α, IL-6, IL-1β, MCP-1, ICAM-1) while upregulating the anti-inflammatory cytokine IL-10. These effects are accompanied by inhibition of the NF-*κ*B signaling pathway, increased NO production and eNOS expression, reduced levels of reactive oxygen species and MDA, and enhanced activity of antioxidant enzymes such as SOD and GSH-Px (94). Collectively, these mechanisms contribute to the attenuation of chronic inflammation and oxidative stress, while improving endothelium-dependent vasodilation and microcirculatory perfusion. Furthermore, in animal models of myocardial ischemia and infarction, Shexiang Baoxin Pill significantly reduced myocardial infarct size, improved left ventricular function, and promoted VEGF-mediated angiogenesis (95, 96).

#### 4.2.2 TXLC: anti-inflammatory, metabolic improvement, and microvascular protection

TXLC alleviates chronic inflammation and immune activation in the context of CHD complicated by DM. Its mechanism involves upregulation of KLF4, inhibition of NF-*κ*B signaling and its downstream inflammatory mediators, alongside improvements in glucose–lipid metabolism and insulin resistance (97–99). In models of diabetic cardiomyopathy and myocardial ischemia/reperfusion, this medicine promotes collateral circulation formation and arteriogenesis, reduces myocardial infarct size or no-reflow areas, improves left ventricular function, and protects endothelial cells by inhibiting pyroptosis and enhancing VEGF-mediated angiogenesis (100–102).

#### 4.2.3 FFDSDP: antioxidant, anti-inflammatory, and anti-myocardial remodeling effects

FFDSDP improves lipid metabolism disorders and stabilizes plaques in high-fat diet-induced and atherosclerosis models. These effects are closely associated with the inhibition of inflammatory responses, attenuation of myocardial remodeling, and prevention of heart failure progression (103, 104). In isoproterenol-induced myocardial ischemia models, this medicine alleviates myocardial injury by enhancing SOD activity, improving myocardial energy metabolism, and increasing microcirculatory perfusion. Furthermore, Danshen and its active constituents have been confirmed to reduce myocardial infarct size and improve cardiac function through antioxidant, anti-inflammatory, and pro-angiogenic mechanisms (104, 105).

#### 4.2.4 YDXNTSC and QSYQDP: endothelial protection and multi-organ microvascular protection

YDXNTSC reduces MDA and ET-1 levels, enhances NO bioavailability, and inhibits NF-*κ*B signaling in hyperlipidemic and myocardial ischemia models, thereby improving endothelial integrity, alleviating oxidative stress, and stabilizing microvascular function (106, 107). QSYQDP demonstrates multi-organ microvascular protective effects in diabetic models by improving renal function, inhibiting Wnt/β-catenin and TGF-β/Smad fibrotic pathways, reducing inflammatory markers, promoting myocardial angiogenesis, and improving left ventricular function (108–111).

#### 4.2.5 GXNT and JLDG: multi-target systemic protection and metabolic regulation

GXNT exerts systemic protective effects along the inflammation–endothelial injury–metabolic disorder–thrombotic risk axis by activating the endothelial NO-cGMP signaling pathway, inhibiting NF-κB-mediated residual inflammation, regulating gut microbiota-associated inflammatory responses, promoting macrophage cholesterol efflux, and suppressing NETs-related thrombosis (112–116). JLDG improves insulin resistance and glucose–lipid metabolism disorders, alleviates multi-organ inflammation, and reduces cardiovascular–renal injury by modulating the PI3K–AKT pathway and inhibiting AGE–RAGE–TNF signaling (117–121).

#### 4.2.6 Other OCPMs: synergistic effects via multiple mechanisms

LWDHP and YXST exert beneficial effects through antioxidant, antithrombotic, endothelial-protective, and metabolic regulatory mechanisms, making them suitable for patients with concurrent hyperlipidemia, endothelial dysfunction, or elevated thrombotic risk (122–126). YXSC and DZJTC exert cardioprotective, anti-inflammatory, and anti-apoptotic effects by modulating oxidative stress-related and NF-*κ*B-dependent signaling pathways, while also improving diabetes-related dyslipidemia and microvascular injury (127–133). FRTMC improves glucose–lipid metabolism, coagulation function, and myocardial fibrosis, supporting its role in myocardial protection under diabetic conditions (134).

Nevertheless, certain discrepancies exist between preclinical studies and actual clinical efficacy, primarily manifested in the following aspects: inherent differences in physiological and pathological characteristics between animal models and humans; variations in monitoring intensity across clinical studies, where differences in medication management between inpatient and outpatient settings may influence efficacy assessment; the indirect impact of social interaction factors such as physician–patient communication and peer support on treatment outcomes through improved psychological status; and considerable variability in patient adherence across different studies. These implementation differences may all influence the actual effectiveness of OCPMs in real-world clinical settings.

### 4.3 Clinical implications

Based on the results of the NMA, this study preliminarily elucidates the relative efficacy differences among various OCPMs combined with conventional Western medicine in patients with CHD complicated by DM from a statistical perspective. It is important to note that the CINeMA evidence quality assessment indicated that the evidence grades for the aforementioned efficacy indicators were generally low, primarily due to limitations related to the risk of bias, imprecision, and heterogeneity among the included studies. Therefore, the findings of this study should only be considered as a preliminary reference based on available statistical results and cannot be used as definitive conclusions to directly guide clinical practice. Nevertheless, based on the current statistical data, the following tiered clinical reference recommendations can be cautiously considered by clinicians, integrating the individual circumstances of each patient.

The first tier comprises preferred references. Shexiang Baoxin Pill combined with conventional Western therapy demonstrates statistically significant advantages in reducing triglycerides, improving left ventricular end-systolic diameter, lowering inflammatory markers (IL-6, TNF-α, hs-CRP), and improving endothelial function (ET-1) and coagulation function (FIB). It is suitable for patients with prominent inflammatory responses, endothelial dysfunction, and high thrombotic risk. Tongxinluo Capsule combined with conventional Western therapy shows the best statistical performance in overall clinical efficacy, reducing 2-h PBG, improving left ventricular end-diastolic diameter, alleviating angina symptoms (reducing weekly attack frequency and duration), and increasing NO levels. It is suitable for patients with significant blood glucose fluctuations, cardiac dysfunction, and frequent angina attacks. From a health economics perspective, although the unit costs of these two medicines are slightly higher, their synergistic improvements across multiple dimensions may offer a better cost-effectiveness ratio and help reduce the number of combined medications.

The second tier comprises references for specific clinical scenarios. Yindan Xinnaotong Soft Capsule shows statistically prominent advantages in increasing left ventricular ejection fraction, reducing whole blood high-shear and low-shear viscosity, and increasing SOD levels. It is suitable for patients with reduced cardiac function, high blood viscosity, and significant oxidative stress. Guanxinning Tablet demonstrates the best efficacy in reducing HbA1c and is suitable for patients with poor long-term blood glucose control. Jinlida Granule performs best in reducing fasting blood glucose and LDL-C, making it appropriate for patients requiring control of both blood glucose and lipids. Liuwei Dihuang Pill shows the best efficacy in reducing the incidence of MACE, suggesting potential long-term preventive value. Yangxinshi Tablet performs best in reducing NT-proBNP, making it suitable for patients with heart failure. Danzhi Jiangtang Capsule shows the best efficacy in reducing TC and is suitable for patients with concurrent hypercholesterolemia. Clinicians may refer to these statistical results when selecting corresponding OCMPs based on a patient's primary clinical issues.

The third tier comprises references for combined application. For patients with multidimensional clinical needs, exploring combination strategies based on the advantages of different OCPMs for specific indicators could be considered, guided by TCM syndrome differentiation. However, caution should be exercised to avoid duplicate medication and potential drug interactions. Future clinical studies are needed to verify the safety and efficacy of such combination therapies.

In summary, the statistical results provide preliminary reference directions for clinical medication selection. However, the above-mentioned recommendations are all based on existing low-level evidence. Clinicians should consider these references in conjunction with the patient's specific condition, individual differences, and drug accessibility. High-quality, multicenter, large-sample RCTs are urgently needed to validate the real-world efficacy of these OCPMs and provide higher-quality evidence-based support for clinical decision-making.

### 4.4 Limitations

This study has the following limitations.

#### 4.4.1 Model fit and risk of bias

Although the NMA model for traditional Chinese medicine interventions demonstrated good model fit, local fluctuations in residuals for some outcomes, potential publication bias in the included studies, and the singularity of diagnostic dimensions remain major limitations that may affect the robustness of the results.

#### 4.4.2 Methodological quality of included studies

The overall methodological quality of the included studies was relatively low, with inadequate reporting of randomization methods and blinding, and large differences in sample sizes between groups, which may introduce selection and measurement bias and affect the accuracy of the conclusions.

#### 4.4.3 Dosage heterogeneity

Dosages of the same OCPMs varied across studies, and subgroup analyses were not conducted, which may interfere with the pooled estimates of efficacy.

#### 4.4.4 Lack of direct comparative evidence

No randomized controlled trials directly comparing different OCPMs were included. All comparisons were indirect, and the number of studies for each medicine was relatively small, which may affect the robustness of the results.

#### 4.4.5 Transitivity and clinical heterogeneity

The reliability of the findings depends on the transitivity assumption of NMA, which requires comparability across studies in terms of clinical and methodological characteristics. The RCTs included in this study all focused on patients with CHD complicated by DM and were all based on conventional Western medicine treatment, providing a consistent clinical background overall. However, there was still variability in treatment duration, disease course, and concomitant medications across studies. These factors may act as potential effect modifiers, thereby influencing the transitivity assumption. Therefore, the results of this study should be interpreted with caution, and future high-quality RCTs with more standardized designs and direct comparisons between interventions are necessary for further validation.

#### 4.4.6 Absence of TCM syndrome differentiation

Although all included RCTs used OCPMs, the majority did not perform TCM syndrome differentiation. According to TCM theory, OCPMs should be prescribed based on specific syndromes (e.g., *qi* and *yin* deficiency, phlegm–stasis intermingling). The pathological characteristics and therapeutic responses to the same OCPMs may differ significantly among patients with different syndromes. However, in current clinical research practice, most trials prioritize Western medical disease diagnostic criteria in their design and fail to strictly adhere to the “treatment based on syndrome differentiation” principle of TCM. This results in a mix of patients with “same disease but different syndromes” within the study population. Consequently, the results of this NMA essentially represent an “all-comers” overall effect estimate based on Western medical disease diagnosis, evaluating the average efficacy of different OCPMs in a population defined by CHD complicated by DM without stratifying by TCM syndrome type. While this analytical strategy reflects, to some extent, the real-world application of OCPMs in routine clinical practice, it may also obscure the heterogeneity of treatment responses among patients with different syndromes, thereby affecting the precision of the conclusions. From a methodological perspective, incorporating all available studies into a single network for comparison—based on current literature—provides a feasible starting point for exploring differences in efficacy among OCPMs. However, it must be acknowledged that the results can only serve as a preliminary reference.

## 5 Conclusions

In summary, this network meta-analysis provides a comprehensive comparative evaluation of commonly used OCPMs in the management of CHD complicated by DM across multiple clinical and biological outcome domains. The findings suggest that combination therapy with OCPMs and conventional Western medicines is generally associated with favorable improvements in glycemic control, lipid metabolism, angina-related symptoms, endothelial function, and selected inflammatory and hemorheological indicators, while differences in efficacy were observed among individual interventions depending on specific outcome measures. These results highlight the potential advantages of multi-target, multi-pathway therapeutic strategies for complex metabolic–cardiovascular comorbidities. Nevertheless, given the heterogeneity in study designs, outcome reporting, and intervention regimens among the included trials, the present findings should be interpreted with caution. Future well-designed, large-scale RCTs with standardized endpoints are warranted to confirm these observations and to clarify the optimal selection and positioning of OCPMs in the integrated management of CHD complicated by DM.

## Data Availability

The original contributions presented in the study are included in the article/Supplementary Material, further inquiries can be directed to the corresponding author/s.
