# Supplementary figures and images for: Comparative efficacy and safety of oral Chinese patent medicines combined with conventional therapy for coronary heart disease complicated by diabetes: a systematic review and network meta-analysis
Source: Front Cardiovasc Med. 2026 Apr 30;13:1785694. doi: 10.3389/fcvm.2026.1785694 (PMC13171381; doi:10.3389/fcvm.2026.1785694)

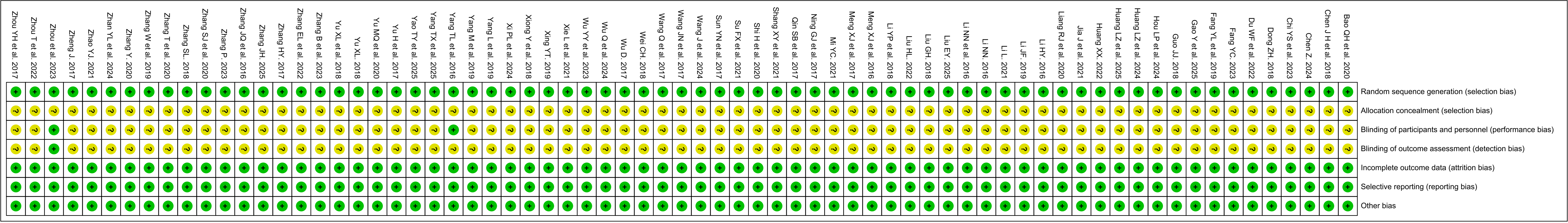

Supplement: Supplementary file 3 [file image1.tif]

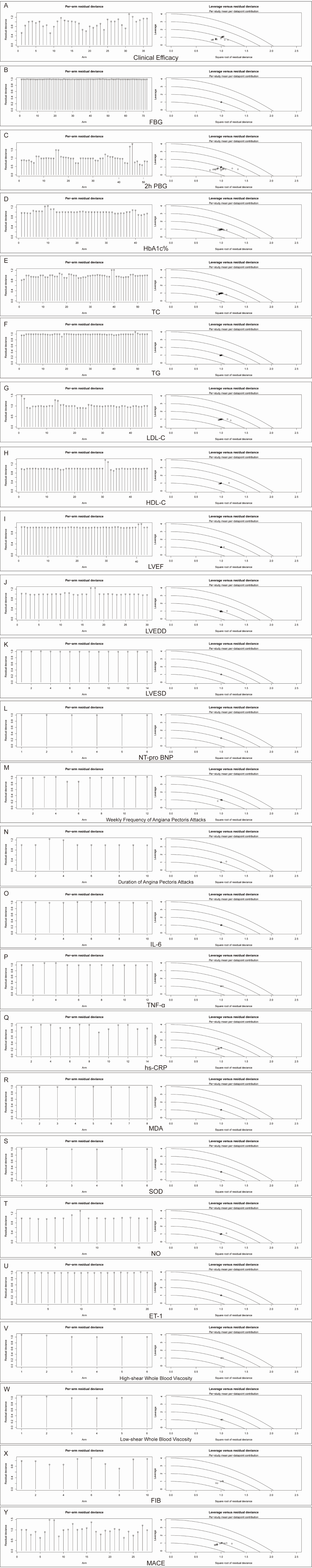

Supplement: Supplementary file 4 [file image2.tif]
